# Detection of Rocio Virus SPH 34675 during Dengue Epidemics, Brazil, 2011–2013

**DOI:** 10.3201/2604.190487

**Published:** 2020-04

**Authors:** Marielena V. Saivish, Vivaldo G. da Costa, Roger L. Rodrigues, Valéria C.R. Féres, Eduardo Montoya-Diaz, Marcos L. Moreli

**Affiliations:** University of Goiás, Jataí, Brazil (M.V. Saivish, R.L. Rodrigues, M.L. Moreli);; University of Brasília, Brasília, Brazil (V.G. da Costa);; Federal University of Goiás, Goiânia, Brazil (V.C.R. Féres);; Paul-Ehrlich-Institut, Langen, Germany (E. Montoya-Diaz)

**Keywords:** Rocio virus, SPH 34675, emerging neurotropic flavivirus, NS5, Brazil, molecular diagnostics, viruses, dengue, vector-borne infections

## Abstract

Recent seroprevalence studies in animals detected Rocio virus in regions of Brazil, indicating risk for re-emergence of this pathogen. We identified Rocio virus RNA in samples from 2 human patients for whom dengue fever was clinically suspected but ruled out by laboratory findings. Testing for infrequent flavivirus infections should expedite diagnoses.

Brazil has been affected by outbreaks caused by viruses of the genus *Flavivirus*, such as dengue (DENV), Zika, and yellow fever viruses, along with co-infections with other arboviruses (*1*). The amino acid sequences of polyproteins from viruses of this genus are very similar, which has limited the development of detection methods, often resulting in cross-reactions within serocomplexes during serologic testing (*2*). Therefore, tracking in areas where mosquito-specific flaviviruses co-circulate may have led to underestimated infections because of the detection and the hierarchy of disease based on medical importance. 

Rocio virus (ROCV) is a potentially emerging neurotropic flavivirus in Brazil; however, because relatively little is known about the biology of this virus, technologies for its detection are limited (*3*–*5*). In 1975, ROCV was found to be related to the causative agent of a fatal outbreak of human encephalitis in Brazil; the case-fatality rate was 13%, and neurologic sequelae affected 20% of patients (*5*). The unexpected outbreak ended in 1980, but little documentation exists with regard to circulation of ROCV in Brazil.

To determine the extent of ROCV circulation in different areas of Brazil, we screened 647 serum samples collected during an outbreak of dengue fever during 2011–2013. The samples came from patients in care units of the public health system, which offer 24-hour outpatient urgent care, and emergency services in the city of Goiânia, central Brazil. The samples were from patients of all age groups and sexes who exhibited signs and symptoms of suspected dengue infection. During the outbreak, the city reported ≈88,000 cases of DENV infection (*6*). Of the 647 samples screened for DENV by use of serologic and molecular methods, 121 were negative for DENV. We subsequently screened those 121 samples for ROCV. 

Using nested PCR with genus-specific primers (*7*), we detected the ROCV nonstructural (NS) 5 gene in 2 of the 121 samples. We used the amplified sequences from the ROCV NS5 gene for phylogenetic analysis, which confirmed 100% identity with the consensus sequence of ROCV NS5 in strain SPH 34675, the strain isolated from the 1975 encephalitis outbreak. Furthermore, the detected NS5 ROCV gene (ROCV 18) is related to Ilheus virus from the Japanese encephalitis virus complex and did not change the topology of the phylogenetic tree with other pathogenic flaviviruses, as previously reported (*3*) (Figure).

**Figure:**
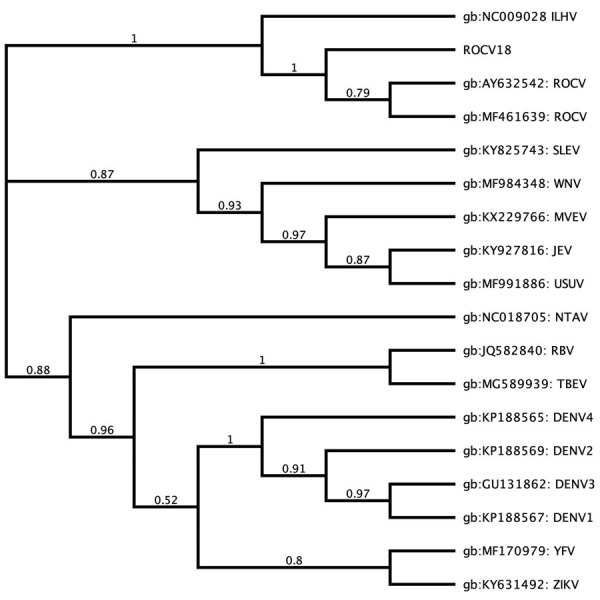
Phylogenetic analysis of the ROCV nonstructural 5 gene (ROCV18) detected during dengue epidemics in Brazil, 2013, and reference sequences. Tree constructed by using the maximum-likelihood method. Pairwise distances were calculated by using the neighbor-joining algorithm, and node numbers represent bootstrap values (10,000 replicates). GenBank accession numbers are provided. DENV, dengue virus; ILHV, Ilheus virus; JEV, Japanese encephalitis virus; MVEV, Murray Valley encephalitis virus; NTAV, Ntaya virus; RBV, Rio Bravo virus; ROCV, Rocio virus; SLEV, Saint Louis encephalitis virus; TBEV, tick-borne encephalitis virus; USUV, Usutu virus; WNV, West Nile virus; YFV, yellow fever virus; ZIKV, Zika virus**.**

The 2 ROCV-positive samples were from a 33-year-old female patient and a 47-year-old male patient. The female patient experienced prostration, abdominal pain, diarrhea, and thrombocytopenia (120,000 platelets/mm^3^), and the male patient experienced headache, eye pain, pruritus, nausea, and leukopenia (3,560 cells/mm^3^) (Appendix). Both patients had fever, myalgia, and arthralgia, but they denied having had chronic diseases and had been vaccinated against yellow fever virus. No information about patients’ residence or travel history was available. The patients received ambulatory care, and their clinical outcome was cure.

The molecular diagnostic result for positive ROCV in humans reported in this study corroborates the results of other studies involving serologic tests for ROCV in animals (*8*,*9*) and demonstrates the high probability that ROCV is circulating in different areas of Brazil. Our findings point out the need for clinicians to clearly establish flavivirus infection diagnoses by testing for various and infrequent regional flaviviruses

Appendix Supplemental methods and results for study of detection of Rocio virus SPH 34675 during dengue epidemics, Brazil, 2011–2013.
