# Supplementary material for: Detection of Rocio Virus SPH 34675 during Dengue Epidemics, Brazil, 2011–2013
Source: Emerg Infect Dis. 2020 Apr;26(4):797–9. doi: 10.3201/2604.190487 (PMC7101124; doi:10.3201/2604.190487)
Supplement: Appendix — Supplemental methods and results for study of detection of Rocio virus SPH 34675 during dengue epidemics, Brazil, 2011–2013. [file 19-0487-Techapp-s1.pdf]

Article DOI: https://doi.org/10.3201/eid2604.190487

Detection of Rocio Virus SPH 34675 during Dengue Epidemics, Brazil, 2011–2013

Appendix

Appendix Table 1. Clinical data of the acute febrile patients positive for Rocio virus infection in Goiânia county, Central Brazil

	Study patients	
Sign or symptom	ROCV (p18), female	ROCV (p21), male
Myalgia	Yes	Yes
Arthralgia	Yes	Yes
Prostration	Yes	No
Abdominal pain	Yes	No
Diarrhea	Yes	No
Headache	No	Yes
Eye pain	No	Yes
Itching	No	Yes
Nausea	No	Yes
Vomiting	No	No
Dizziness	No	No
Epistaxis	No	No
Ascites	No	No
Palpebral edema	No	No
Neurological	No	No
Exanthema	No	No
Shaking	No	No
Lethargy	No	No
Paresthesia	No	No
Paresis	No	No
Jaundice	No	No
Cyanosis	No	No
Hepatomegaly	No	No
Splenomegaly	No	No
Cholecystitis	No	No
Respiratory distress	No	No

Appendix Table 2. Results of laboratory tests of acute febrile patients positive for Rocio virus in Goiânia county, Central Brazil*

	Study patients					
	ROCV (p18)			ROCV (p21)		
Laboratory tests	T1	T2	T3	T1	T2	T3
Hematocrit (%)	32.7	36.7	34.9	47.4	46.1	45.8
Hemoglobin (g/dL)	10.8	11.9	11.4	15.5	15.4	16.6
Leukocyte (cells/mm3)	6,900	4,900	4,500	3,560	3,400	3,730
Platelets (cells/mm3)	120,000	220,000	147,000	207,000	201,000	198,000
AST (mg/dL)	17	19	19	35	42	29
ALT (mg/dL)	12	78	15	31	51	28
Albumin (mg/dL)	3.4	3.6	3.6	4.3	3.83	4.2
Creatinine (mg/dL)	1.1	1.06	1.05	1.08	1.12	1.19
Direct bilirubin (mg/dL)	0.18	0.27	0.16	0.16	0.29	0.26
Indirect bilirubin (mg/dL)	0.22	0.23	0.24	0.51	0.6	0.62
Activated partial thromboplastin time (seconds)	79.2	76.6	49.9	72.6	48.2	100
International normalized ratio (%)	1.1	1.12	1.38	1.19	1.57	0.53

*T1, sample collected until the seventh day of postdisease onset (PDO); T2, sample collected between the eighth and 15th day PDO; T3, sample collected between the 20th and 30th day PDO.

Appendix Table 3. Patient data and background information for the two samples positive for Rocio virus in Goiânia county, Central Brazil

Patient data/background	Study patients	
	ROCV (p18)	ROCV (p21)
Type of patient	Ambulatory patient	Ambulatory patient
Year of blood sample collection?	2012	2013
Has the patient ever had yellow fever?	No	No
Has the patient ever been vaccinated against yellow fever?	Yes	Yes
Has the patients ever had dengue?	-	Yes (2008)
Diabetes mellitus?	No	No
Chronic renal failure?	No	No
Asthma?	No	No
Lupus	No	No
Cancer?	No	No
AIDS?	No	No
Has the patient had hepatitis B or C?	No	No
Has the patient ever had a transplant?	No	No
Does the patient use any medication regularly?	-	No

Appendix Table 4. Results of laboratory tests for DENV in Goiânia county, Central Brazil*

Laboratory tests		Study patients					
		ROCV (p18)			ROCV (p21)		
		T1*	T2†	T3‡	T1	T2	T3
ELISA	IgM anti-DENV	NR[d]	NR	NR	U[e]	U	-
			OD[f]= 0.26			OD= 0.3	
	IgG anti-DENV	NR	NR	NR	R[g]	-	-
			OD= 0.15			OD= 2.1	
NS1 DENV		NR	-	-	NR	-	-
RT-PCR		Negative	-	-	Negative	-	-

*OD, optical density; NR, not reactive; R, reactive; T1, Sample collected until the seventh day after disease onset (ADO); T2, sample collected between the eighth and 15th day ADO; T3, sample collected between the 20th and 30th ADO; U, undetermined.

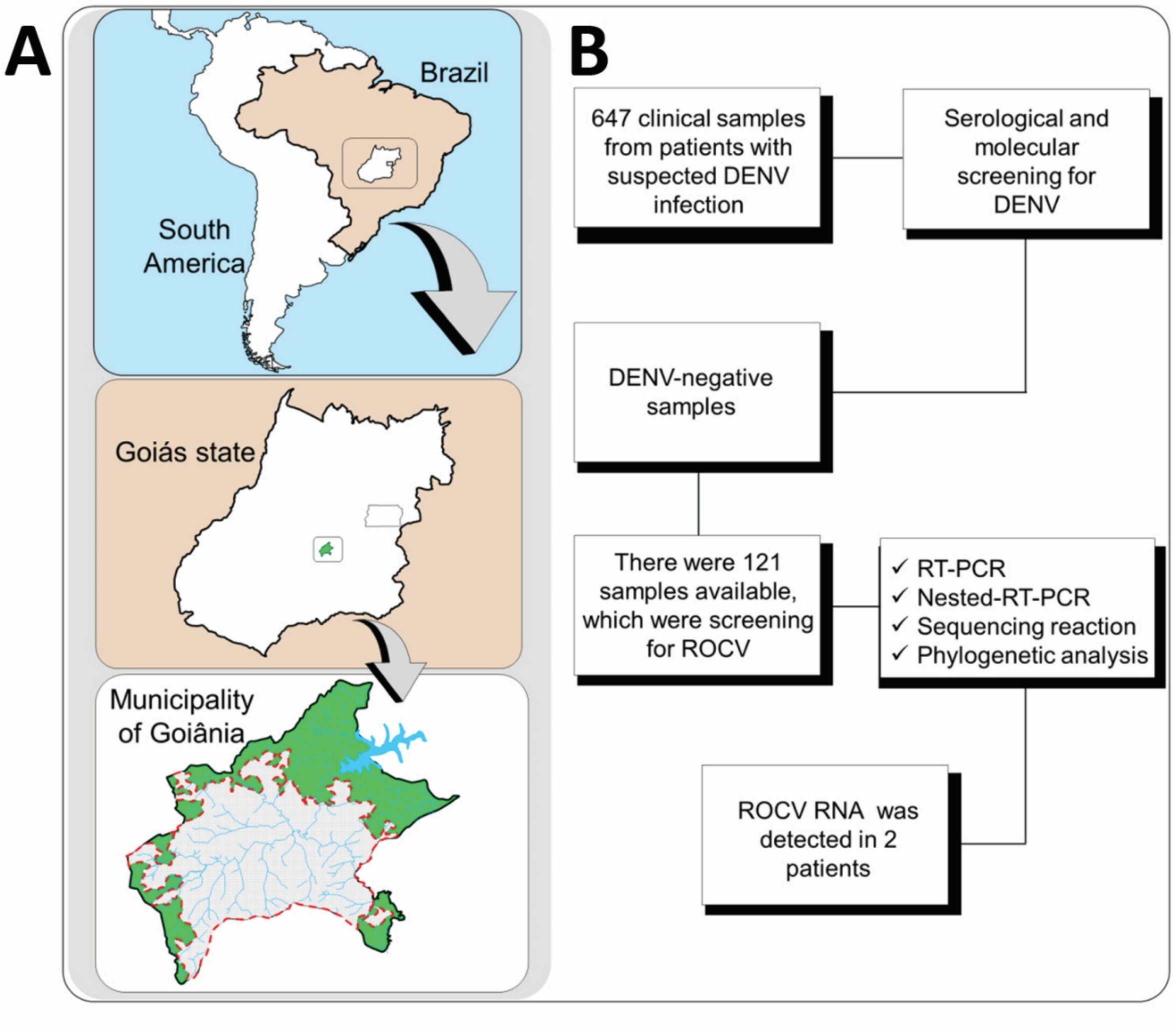

Appendix Figure. Map of the study site and methodology flowchart. Panel A shows a map of Goiás state (Brazil) with the location of the municipality of Goiânia. Panel B shows the methodology flowchart.
